# Supplementary figures and images for: A meta-analysis of transcriptomic profiles of Huntington’s disease patients
Source: PLoS One. 2021 Jun 10;16(6):e0253037. doi: 10.1371/journal.pone.0253037 (PMC8191979; doi:10.1371/journal.pone.0253037)

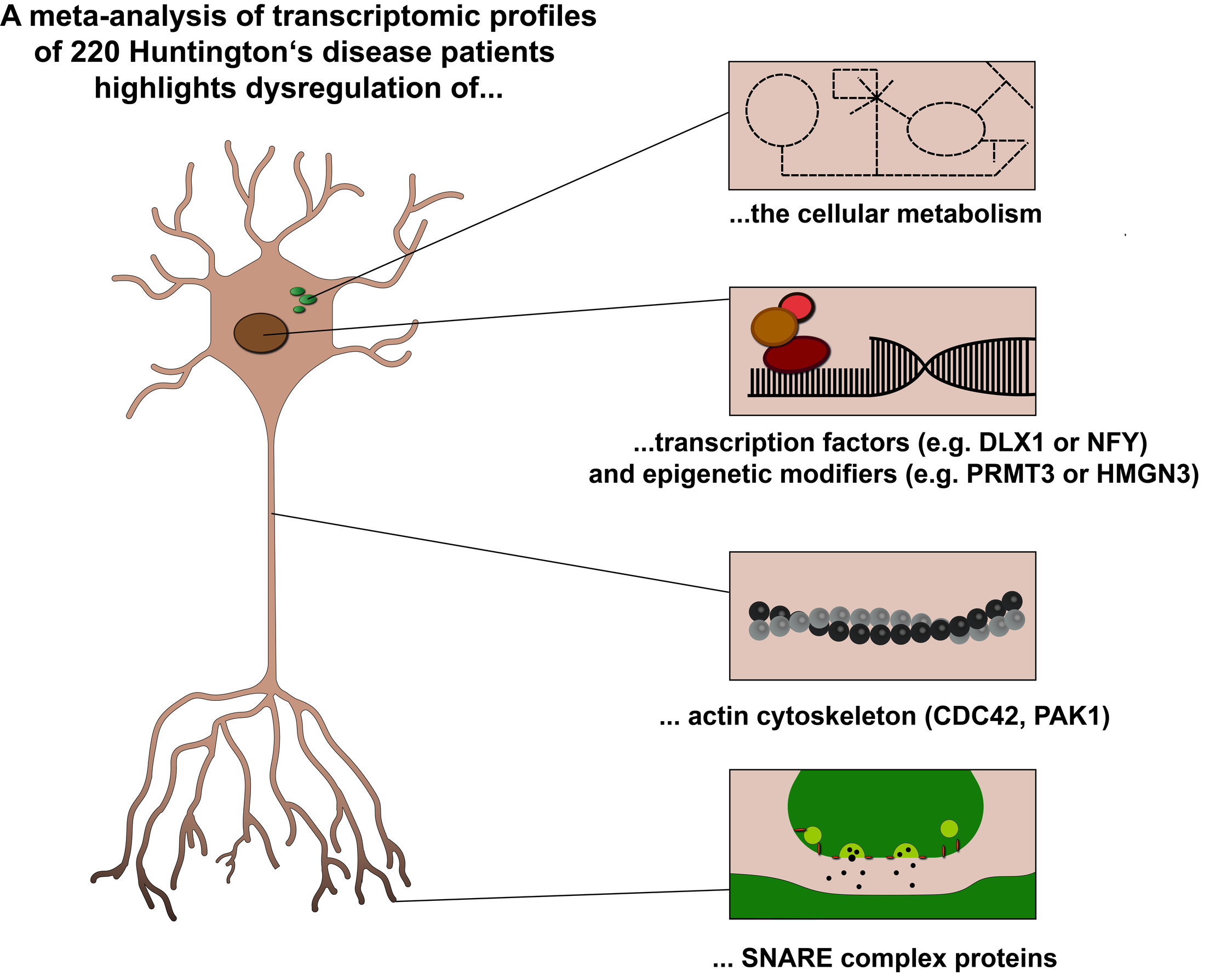

Supplement: S1 Fig — (TIF) [file pone.0253037.s001.tif]

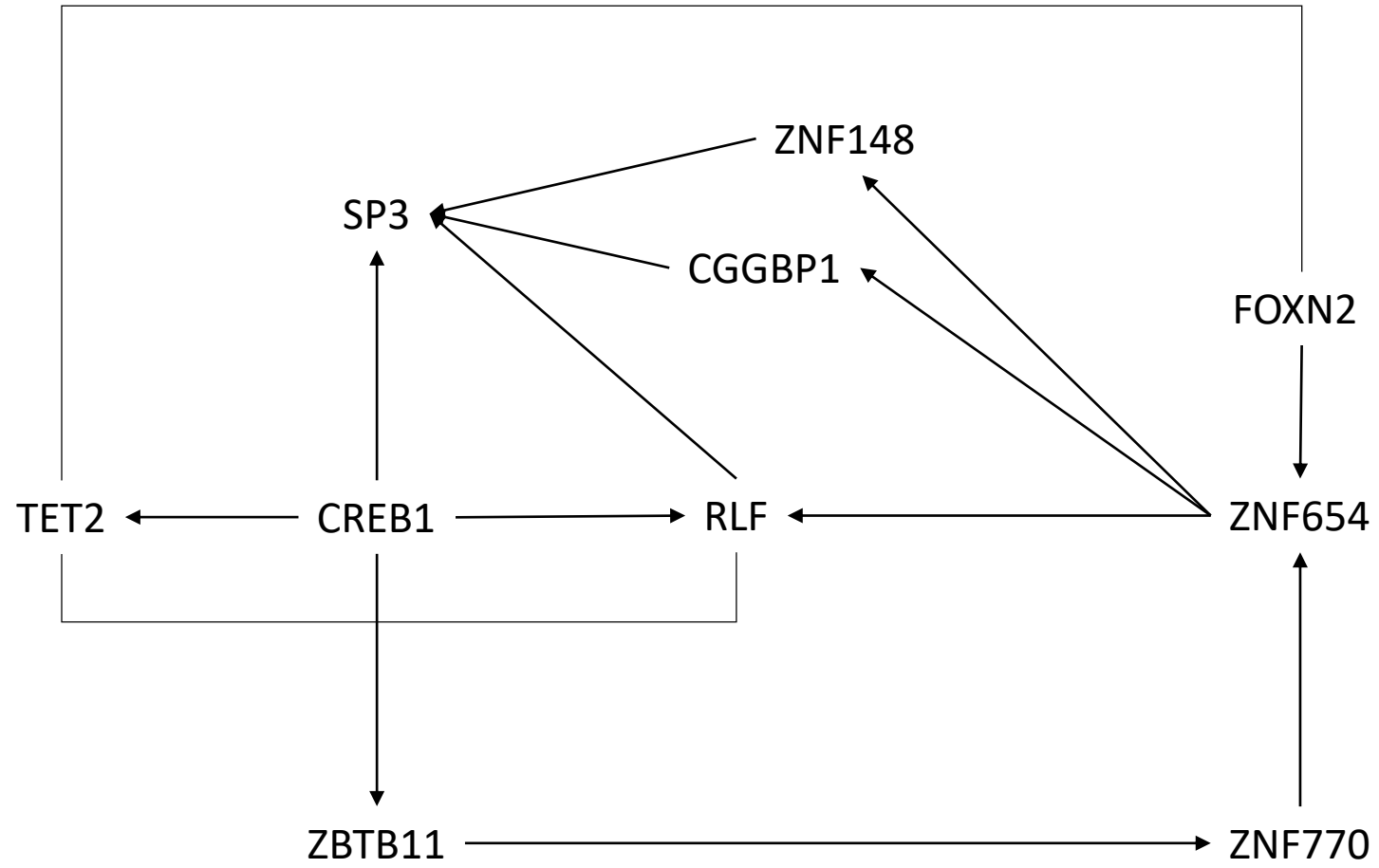

Supplement: S8 File — (PDF) [file pone.0253037.s009.pdf]

# HMG3

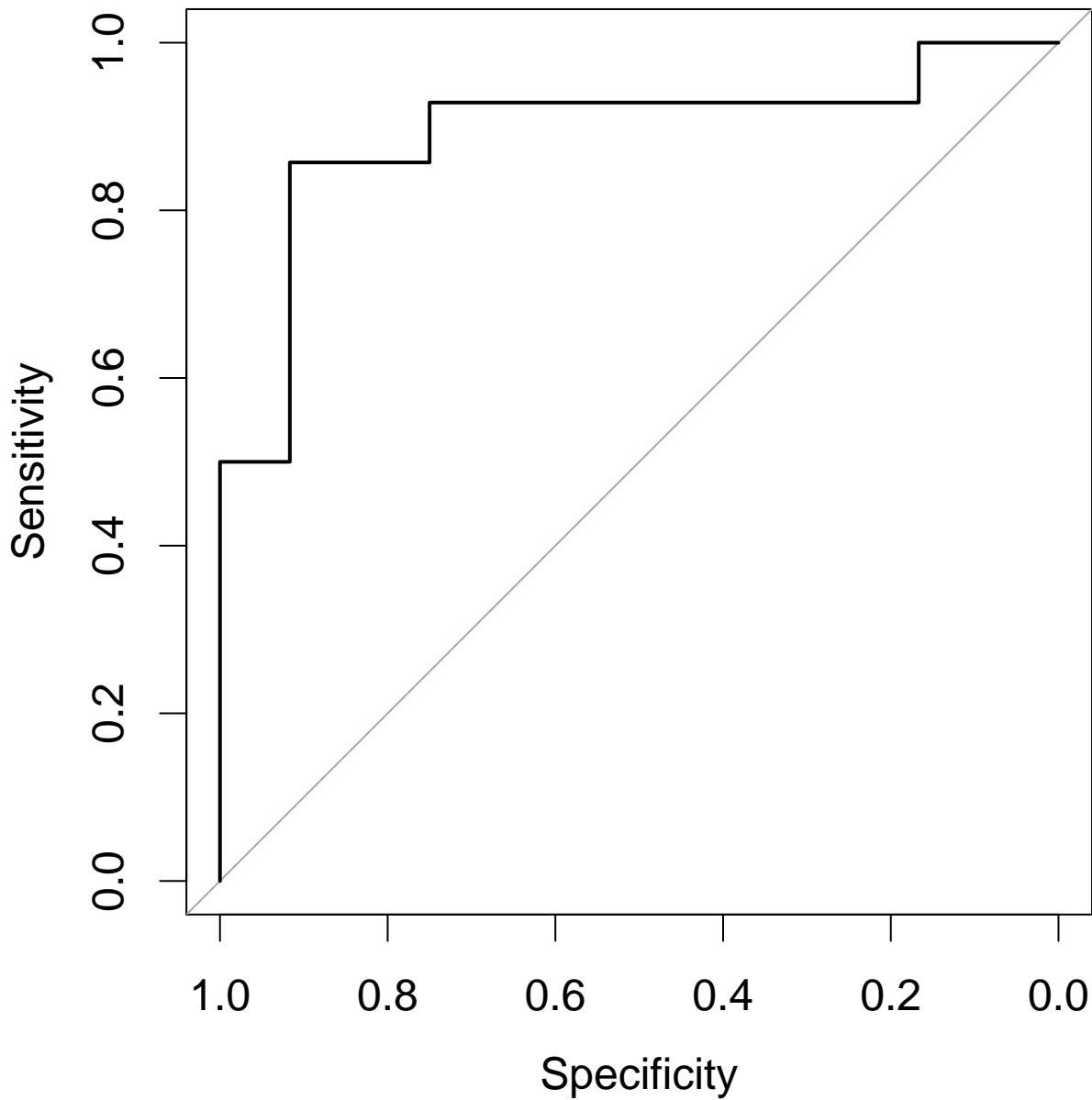

# CDC42

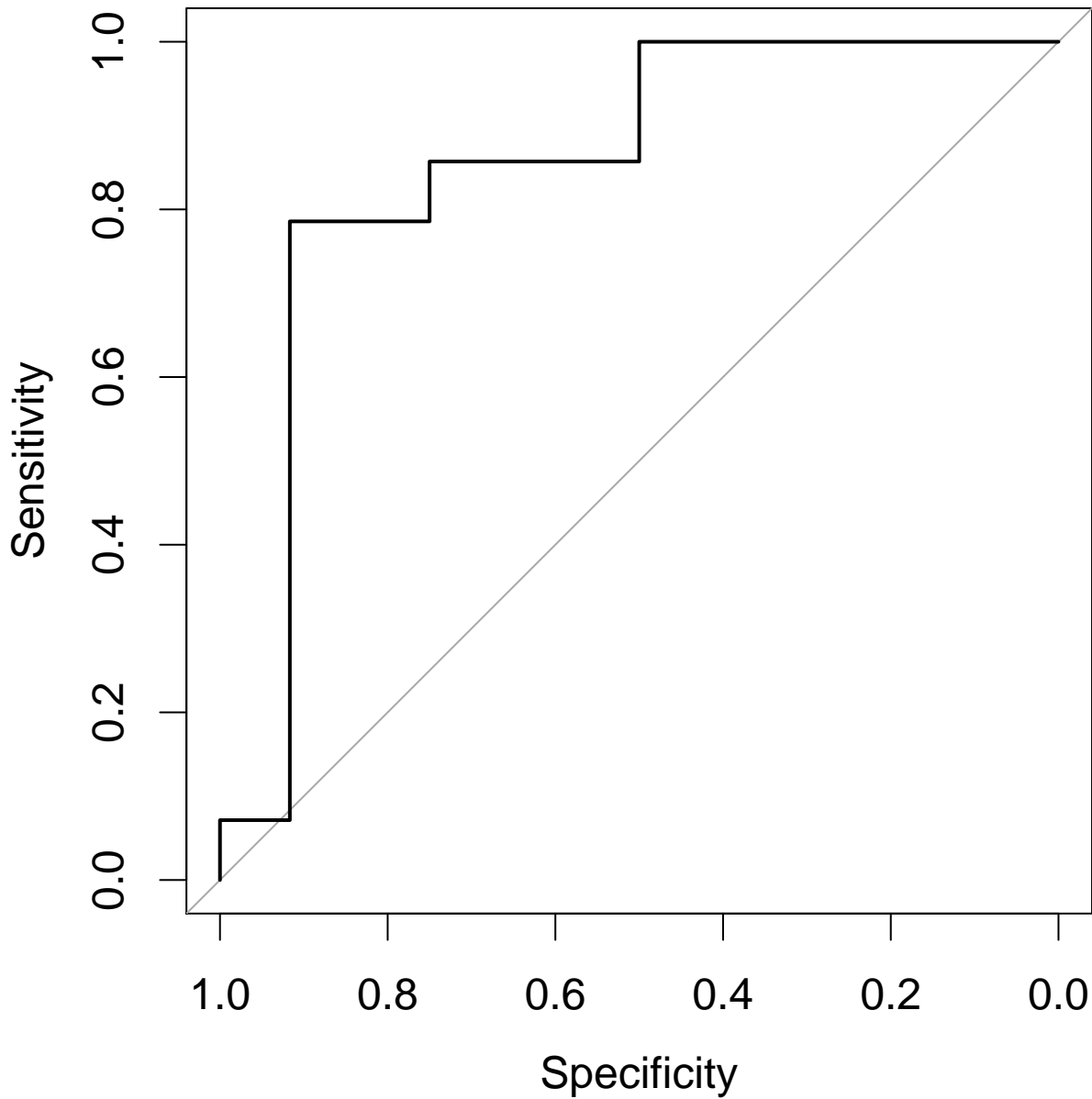

# DNMT3A

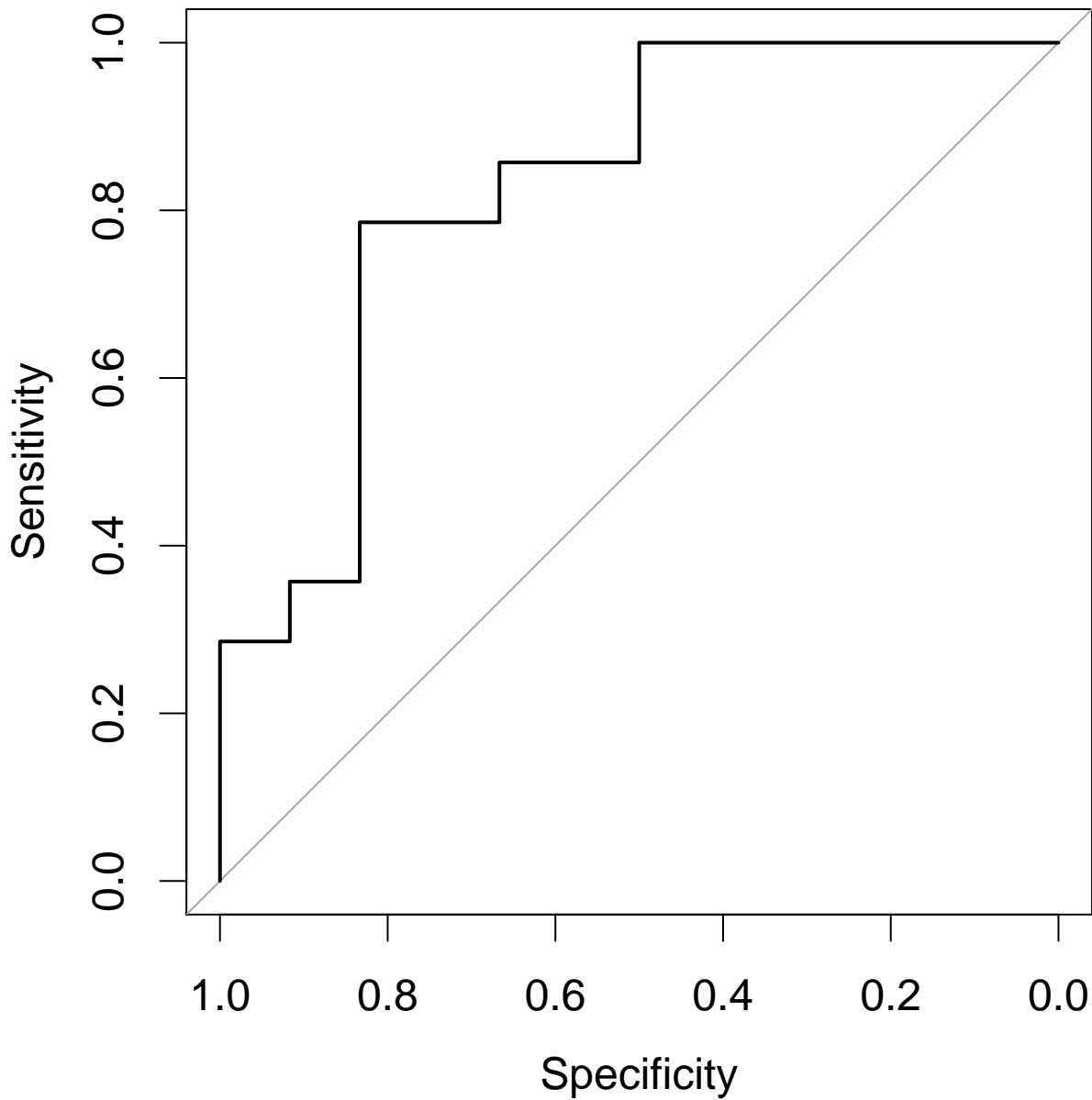

# NFYA

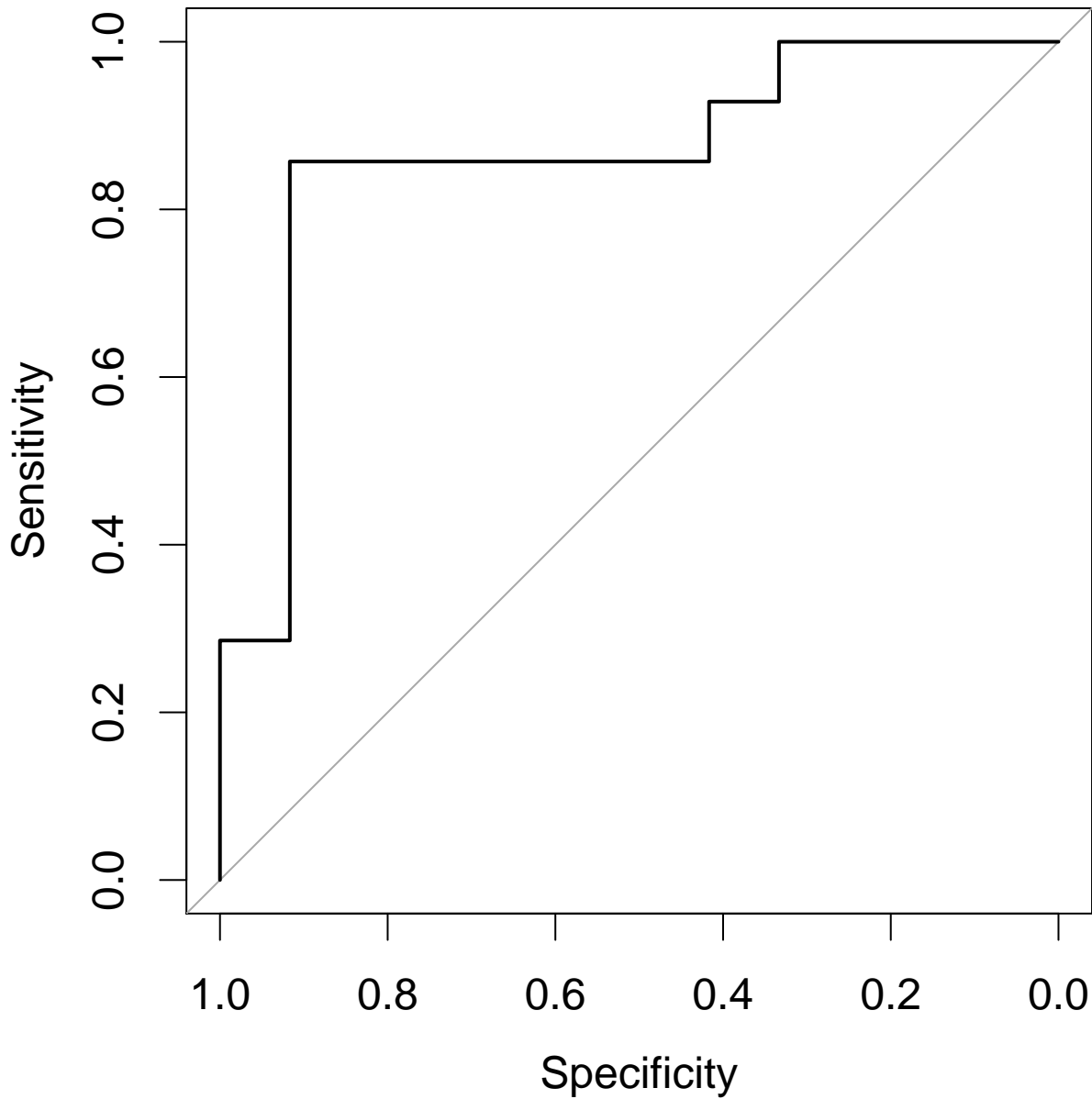

# NFYB

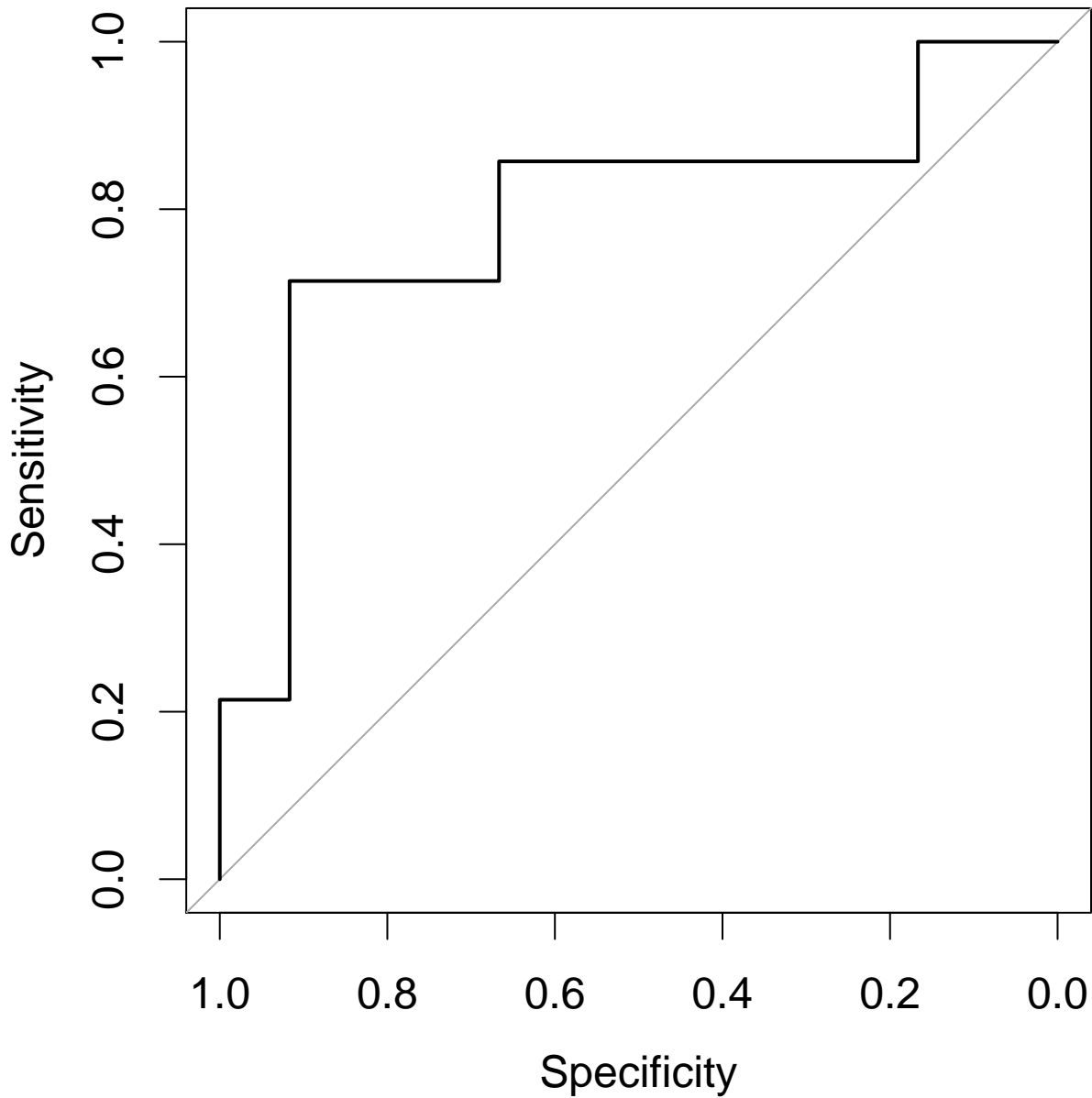

# VAMP7

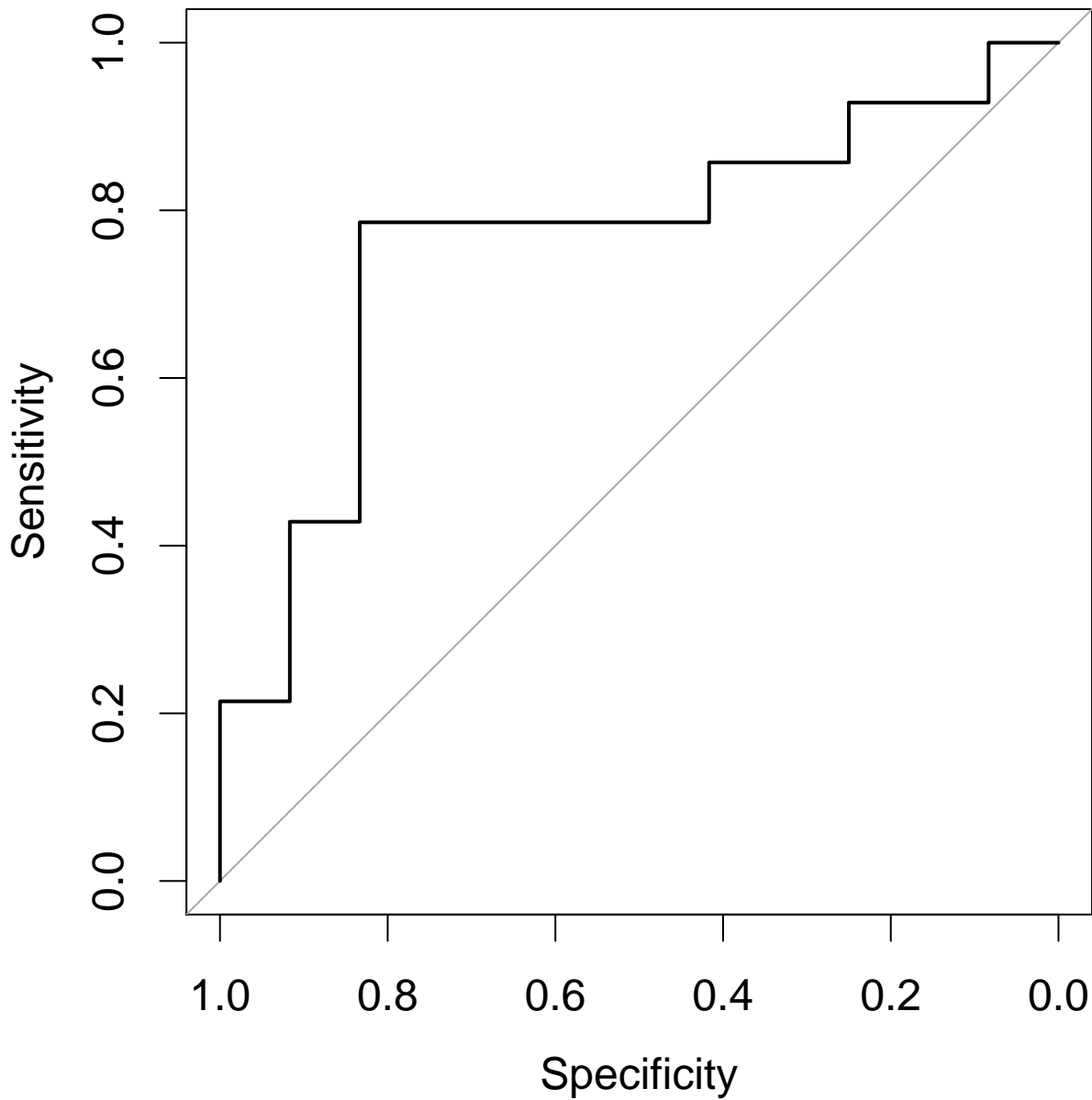

Supplement: S11 File — (PDF) [file pone.0253037.s012.pdf]
