# Supplementary material for: A meta-analysis of transcriptomic profiles of Huntington’s disease patients
Source: PLoS One. 2021 Jun 10;16(6):e0253037. doi: 10.1371/journal.pone.0253037 (PMC8191979; doi:10.1371/journal.pone.0253037)
Supplement: Attachment — Submitted filename: Response.docx [file pone.0253037.s013.docx]

Abteilung Gentherapie

Manuel Seefelder, M.Sc.

Helmholzstraße 8/1

89081 Ulm, Germany

Tel: +49 731 500-46112

manuel.seefelder@uni-ulm.de

06th April 2021

**Universität Ulm |** 89069 Ulm | Germany

PLOS ONE

Editorial Office

Manuscript PONE-D21-03462: “A meta-analysis of transcriptomic profiles from Huntington’s disease patients points to a pathophysiological role of CDC42, NFY, DLX1 and PRMT3”

Dear Professor Lu,

we thank you for considering our manuscript for publication in PLOS ONE and thank the two reviewers for their careful evaluation of the manuscript, their constructive critic, and their thoughtful suggestions.

In the following, we address the comments raised by you and the two reviewers in detail:

**Editor / Editorial Office:**

- ***“Please ensure that your manuscript meets PLOS ONE's style requirements, including those for file naming.”***

We checked again whether the manuscript meets all style requirements and made some changes in referencing the supporting information and file naming.

- ***“We note that you have included the phrase “data not shown” in your manuscript. Unfortunately, this does not meet our data sharing requirements. PLOS does not permit references to inaccessible data. We require that authors provide all relevant data within the paper, Supporting Information files, or in an acceptable, public repository. Please add a citation to support this phrase or upload the data that corresponds with these findings to a stable repository (such as Figshare or Dryad) and provide and URLs, DOIs, or accession numbers that may be used to access these data. Or, if the data are not a core part of the research being presented in your study, we ask that you remove the phrase that refers to these data.”***

We have removed the phrase “data not shown” in lines 114 and 369 since the database containing the mentioned information has been already cited in the same sentence.

- ***“ We note that you have indicated that data from this study are available upon request. PLOS only allows data to be available upon request if there are legal or ethical restrictions on sharing data publicly. For more information on unacceptable data access restrictions, please see http://journals.plos.org/plosone/s/data-availability#loc-unacceptable-data-access-restrictions.”***

We have now uploaded the R code to a GitHub repository that is publicly accessible under the URL <https://github.com/ma-seefelder/HD_meta_analysis>. Henceforth, we would like to request a change in the statement on data availability from “R scripts used for the analysis are made available upon reasonable request to the corresponding author” to “ R scripts used for the analysis can be retrieved from the GitHub repository under the following URL https://github.com/ma-seefelder/HD_meta_analysis”.

**Reviewer 1**

- ***“RRA analysis is the basis of such bioinformatic study on existing RNA-seq datasets, the authors need to show the RRA score < 0.05 is stringent enough for this study. For example, one of the top hits identified in this paper, PAK1, its expression varied a lot in 3 datasets used here (Z=2.07, -0.42, 0.30), while PAK3 (Z=1.98, 0.46, 0.70) was not identified by RRA, such results are puzzling. The authors need to explain whether they added any weights to certain dataset(s). Other similar research often used RRA score <0.01 as the threshold, some even applied the leave one out cross-validation on the RRA algorithm to get more stable and trustworthy p-values.”***

We agree with the reviewer’s comment that the selection of the RRA score is crucial for the results of any study using robust rank aggregation analysis. Concerning the reviewer’s recommendation of using leave-one-out cross-validation on the RRA algorithm, we believe that this cannot be applied here because only three different studies were compared. In our opinion, an RRA score threshold of 0.05 is the optimal compromise between stringency and sensitivity. An RRA score threshold of 0.05 has been also previously frequently used by others (Song et al. Aging, 2019; Gholaminejad et al., Heart Failure Reviews, 2021; Zare et al., Int J Mol Cell Med., 2019; Jun A et al., Cancers, 2021; Zhang et al., J Cell Mol Med, 2021). Moreover and importantly, we applied RRA only for the initial screening of genes with altered mRNA levels and combined it with WGCNA analyses. The subsequent WGCNA analysis would further exclude genes that did not correlate with HD.

Concerning the “puzzling result” of PAK1 and PAK3, it should be noted that the RRA score of PAK1 is at the edge of significance with a p-value of 0.049, whereas the PAK3 only has a slightly higher RRA score of 0.1. Further, the presented results of altered PAK1 mRNA levels in YAC128 (Z-ratios: 1.47, 2.08) further substantiates the likely relevance of alterations in PAK1 mRNA levels in PAK1. The extensive discussion of PAK1 and other identified hits in the manuscript was not only based on the robust rank aggregation analysis but also the other bioinformatical analyses (WGCNA, protein interaction networks, murine data).

- ***“The authors compared their finding in HD patients to two HD mouse models: R6/2 and YAC128. The latter is known for having very few transcriptional deficits, even at 2-yr old. People have performed a more sophisticated transcriptional study in HD knock-in mice series and identified CAG-length and age-dependent gene expression deficits in multiple tissues (Langfelder et al., Nat Neurosci. 2016). This dataset should be used to confirm the key hub gene expressions (DLX-1, PRMT3 etc.).”***

Following the reviewer’s suggestion, we now have added this dataset to our meta-analysis and added the corresponding data to supplementary file 9. In these datasets, the change in mRNA levels of NFYA, HMGN3, and PAK1 strongly correlate with the polyQ length of the knocked-in *HTT* gene. CDC42 mRNA levels were also elevated in these mice but the correlation with the polyQ length was less and only visible in 10-month old mice (line 399)

- ***“The quality of the figures need to be improved: Figure 1, 2 and 6 has minimal information, as the authors didn’t show how many genes in each module or meta-module, how they distribute among HD and control samples (using module eigengenes), which are the top genes. All these can and should be added to the figures. Also, in Figure 5 and 8 (String data), hub gene and its directly connected nodes (e.g. CDC42 and its connectors) can be coloured differently in STRING database, to make them more noticeable.”***

Based on the reviewer’s comments we have modified figures 1, 2, and 6 and have added the number of genes and the two hub genes with the highest intramodular connectivity for each module. Concerning the reviewer’s comment on the distribution among HD and control samples, we have added a further explanatory sentence to the caption describing that a positive correlation score in the heatmap shows that mRNA levels of genes from the respective module are elevated in samples from HD patients. For figure 5 and 8, we coloured the hub genes and their direct connectors differently.

- ***“The authors mentioned “Strikingly, alterations of mRNA levels of these genes were mostly reciprocal between HD and AD or PD, i.e. genes with an elevated mRNA level in the brain of HD patients showed decreased mRNA levels in the brain of AD or PD patients”. Please add data and discussion about this point.***

Data to this comment have been added to the supplementary files (S7). Based on the reviewer’s r comment, an additional reference to the supplementary table containing the data was added. Moreover, we added a paragraph discussing the observation (lines 588).

- ***Another paper by Moss et al. (Scientific Reports, 2017) performed RNA-seq in HD patients’ brains and blood and showed HD shared some common signatures with AD. This dataset should be added to this study if possible, or at least be compared to the current data and discussed the similarities and differences.”***

Moss et al. 2017 (Scientific Reports, doi: 10.1038/srep44849) described that in an Alzheimer’s disease brain the transcriptional signature overlapped with a transcriptional signature that they identified in the blood of HD patients. In contrast to the present meta-analysis, Moss et al. did not compare transcriptional signatures in the brain of HD patients with changes in the brain of AD patients. As described in the manuscript, we did not observe a large overlap of transcriptional changes between blood and brain of HD patients.

We believe, that the comparison of transcriptional signatures between AD and HD in the study of Moss et al. and this meta-analysis would not lead to significant insights into the transcriptopathy of HD or other neurodegenerative diseases in the brain.

**Reviewer 2**

- ***“However, there are so many analyses performed here that the manuscript as written is hard to follow, thus considerable revision is needed. This work should be revised in terms of how the data is presented as far as 1) additional subheadings, 2) grouping of the analyses in a way that allows the reader to follow more clearly the approaches being taken and why they are taken (TFEA analyses and GO analyses, STRING databases, GeneMania, etc.).”***

Based on the reviewer’s comment, we have added l subheadings in the manuscript to improve its clarity and structure (e.g. lines 71, 140f., 172f., 215, 255, 266, 291f., 299, 321). Further, we now explain the reasons behind each bioinformatical approach in more detail (e.g. lines 142ff. and 174ff.)

We believe that in this revision the more detailed structure of the manuscript and additional explanations for the reasons behind different approaches strongly improved the comprehensibility of the manuscript.

- ***“It should be clear why the M1 network is focused on in the work”***

We added a more concise explanation of why the M1 network is focused on the work (lines 136ff.). The main criterium for our decision was the absence of a positive and significant correlation between gene significance (GS) and module membership (MM) of genes belonging to M2 and M3 as described in lines 115ff and figure 2A. A negative correlation between GS and MM, as for M3 indicates that genes that are more central in the respective WGCNA module are less correlated with, in this case, HD.

We believe that we have addressed all comments of the Reviewers. We would like to resubmit this revised version of the manuscript for further evaluation.

Sincerely,

Manuel Seefelder and Stefan Kochanek
