# Supplementary material for: A meta-analysis of transcriptomic profiles of Huntington’s disease patients
Source: PLoS One. 2021 Jun 10;16(6):e0253037. doi: 10.1371/journal.pone.0253037 (PMC8191979; doi:10.1371/journal.pone.0253037)
Supplement: Attachment — Submitted filename: Response_2.docx [file pone.0253037.s014.docx]

Abteilung Gentherapie

Manuel Seefelder, M.Sc.

Helmholzstraße 8/1

89081 Ulm, Germany

Tel: +49 731 500-46112

manuel.seefelder@uni-ulm.de

25th Mai 2021

**Universität Ulm |** 89069 Ulm | Germany

PLOS ONE

Editorial Office

Manuscript PONE-D21-03462: “A meta-analysis of transcriptomic profiles of Huntington’s disease patients”

Dear Professor Lu,

we thank you for considering our manuscript for publication in PLOS ONE and thank the two reviewers for their careful evaluation of the manuscript, their constructive critic, and their thoughtful suggestions.

In the following, we address the comments raised by you and the two reviewers in detail:

- ***Please include gene numbers and top hub genes for each module in Fig 1a, just like in Fig 2 and 6. 2.***

Figure 1 has been also updated with the information on gene numbers and hub genes in each module.

- ***The authors identified and discussed quite a few genes and related pathways in the manuscript, but selected 4 genes to put in the title. A general title without gene names or just emphasizing CDC42 is more suitable.***

The title of the manuscript was changed in response to your comment. We decided on the more general title “A meta-analysis of transcriptomic profiles of Huntington’s disease patients”.

We believe that we have addressed all comments of the Reviewers. We would like to resubmit this revised version of the manuscript for further evaluation.

Sincerely,

Manuel Seefelder and Stefan Kochanek
